# Till Death (Or an Intruder) Do Us Part: Intrasexual-Competition in a Monogamous Primate

**DOI:** 10.1371/journal.pone.0053724

**Published:** 2013-01-23

**Authors:** Eduardo Fernandez-Duque, Maren Huck

**Affiliations:** 1 Department of Anthropology, University of Pennsylvania, Philadelphia, Pennsylvania, United States of America; 2 Centro de Ecología Aplicada del Litoral, Conicet, Argentina; Texas A&M University, United States of America

## Abstract

Polygynous animals are often highly dimorphic, and show large sex-differences in the degree of intra-sexual competition and aggression, which is associated with biased operational sex ratios (OSR). For socially monogamous, sexually monomorphic species, this relationship is less clear. Among mammals, pair-living has sometimes been assumed to imply equal OSR and low frequency, low intensity intra-sexual competition; even when high rates of intra-sexual competition and selection, in both sexes, have been theoretically predicted and described for various taxa. Owl monkeys are one of a few socially monogamous primates. Using long-term demographic and morphological data from 18 groups, we show that male and female owl monkeys experience intense intra-sexual competition and aggression from solitary floaters. Pair-mates are regularly replaced by intruding floaters (27 female and 23 male replacements in 149 group-years), with negative effects on the reproductive success of both partners. Individuals with only one partner during their life produced 25% more offspring per decade of tenure than those with two or more partners. The termination of the pair-bond is initiated by the floater, and sometimes has fatal consequences for the expelled adult. The existence of floaters and the sporadic, but intense aggression between them and residents suggest that it can be misleading to assume an equal OSR in socially monogamous species based solely on group composition. Instead, we suggest that sexual selection models must assume not equal, but flexible, context-specific, OSR in monogamous species.

## Introduction

The relationships between sexual selection, aggression, operational sex ratio (OSR, the ratio of fertilizable females to sexually active males at any given time), sexual dimorphism, mating system, and population structure are well recognized [1]–[5]. For example, as the operational sex ratio becomes more biased, competition and aggression increase [1], [2], [6]. Generally, polygynous species are more sexually dimorphic than monogamous ones, and sexual dimorphism in body and canine size increases with increased competition in polygynous species [7]–[9]. Although the mating system can only partly explain the strength of intra-sexual competition [7], it has been assumed that species with a monogamous mating system will have similar OSRs given that there is only one reproducing male and female in each group [1]. The potential reproductive rate, however, will also be influenced by parental investment, which can bias the OSR towards the less caring sex [5], [6], [10]. Additionally, for some mammals, and particularly primates, monogamy has been assumed to imply low frequency, low intensity intra-sexual competition [7], [11], [12], whereas more pronounced intra-sexual competition is predicted when there are stronger deviations from an equal OSR [2], [5]. Nevertheless, high rates of intra-sexual selection and competition among both males and females of monogamous species have been theoretically predicted, and have been described for various taxa [6], [12]–[15].

Owl monkeys are one of the few pair-living and genetically monogamous mammals [16], [17, Huck, Babb, Schurr, & Fernandez–Duque, unpubl. data]. They live in small social groups that include one pair of reproducing adults. They show little sexual dimorphism in morphological, behavioural and life-history traits [18], [19]. Males are prominently involved in infant care and nursing and carrying of the infant by both parents last approximately six months [20]–[23]. Given that parents are both strongly involved in infant care, that reproduction is seasonal and that owl monkeys reproduce once a year, potential reproductive rates ( i.e., “times in” and “times out”, sensu [6]) for males and females are the same. In other words, at a population level, they have an equal adult sex ratio [21] and therefore the population-wide OSR can be considered un-biased [5]. Within-group aggression is rare, and although interactions with neighbouring groups can be intense, they do not normally include physical contact. On the other hand, groups routinely interact, sometimes aggressively, with a significant number of solitary “floating” individuals who range over a wide area rather than a fixed territory after having dispersed from their natal groups, and are usually sub-adults or young adults (ca. 3–5 years old) [24].

We present the results of an examination of the relationship between pair-bond duration, intra-sexual competition, and OSR in a population of pair-living Azara's owl monkeys (*Aotus azarai*) in the Argentinean Chaco. Comparing the reproductive success of individuals with one or more partners, we first evaluated the hypothesis that the break-up of pairs is an adaptive strategy for the remaining pair-mate, as is the case in various birds [25]–[27]. We then examined potential reasons for pair-bond termination, which can include both intrinsic (e.g., incompatibility of mates) and extrinsic factors (e.g., solitary individuals) to the pair [26]. Finally, since the relationship between intra-sexual aggression in both sexes and the mating system are contradictory for socially monogamous species that are generally assumed to have an un-biased OSR (see above), we also discuss the term OSR, based on the system found in owl monkeys.

## Materials and Methods

Owl monkey groups have been monitored regularly at the Estancia Guaycolec, Formosa, Argentina (58°11′W, 25°58′S) since 1997. During these 16 years, we collected demographic data from 18 groups. Thus, we were able to establish replacement dates often to the exact date and sometimes within a range of a few weeks [28]. Animals (n = 154) have been captured regularly since 2000 for a complete physical exam and fitted with radio or bead collars for individual identification [29], [30]. We used here only data on identified pairs. We considered cases where a former pair-mate died or disappeared before a new individual was seen in the group as deaths. If a new individual was seen in the group before the former resident disappeared, or if the former resident was later found to range solitary, the replacement was considered an expulsion. We used survival analysis with Weibull distribution [31] and we considered censored data to determine individual or pair tenure lengths.

To evaluate the hypothesis that the break-up is an adaptive strategy for at least one of the original partners, we determined for each individual the number of partners and the number of offspring they had, and calculated the number of infants per 10 years of tenure length (infants/10ytl) or pair length (infants/10ypl). We compared the number of infants/10ytl for individuals that had only one with those that had two or more partners using a general linear model (after inspecting visually for deviations from normality) with number of partners (one or more) and sex as fixed factors. Since any effect found in that analysis could be related to the duration of pair-bonds, we used a linear mixed effect model with female and male identity as random factors to investigate the relationship between duration of a pair and the number of infants/10ytl. We checked the suitability of the model using graphical methods [32], and we ran a Shapiro-Wilk test to confirm that there was no statistically significant deviation from normality in the residuals (W = 0.97, p = 0.64). During the physical exam of individuals we scored wounds at the ears on a scale from 0 (no wounds) to 3 (badly damaged ears), and noted the number of scars, missing teeth and broken canines. We compared the scores of adult animals who were still resident in a breeding group (‘residents’) with those of adults that had been expelled from their group in two ways: using a Mann-Whitney U-test, and matching five residents with five expelled individuals of similar age in a Wilcoxon matched-pair test. Matched individuals had no more than two months of age difference and the mean age for both residents and expelled individuals was 103 months. All analyses were two-tailed and conducted in R 2.13 [33]. We used package lme4 for the mixed effect model [34].

## Results

Owl monkeys did not pair for life; both males and females were regularly replaced by intruding adult individuals, with both males and females being replaced equally often. The intruders had been floaters, relatively young adults who had dispersed from their natal groups. We recorded 27 female and 23 male replacements (including ‘double replacements’ when occasionally two or three replacements occurred in quick succession) during 149 group-years in 18 groups between 2001 and 2011 (G-test with William's correction, G_corr_ = 0.32, df = 1, p = 0.57). Median pair-duration of 26 pairs that stayed long enough together to produce at least one offspring was 9.1 years (survival analysis taking censored data into account), and individuals had a median number of two partners during their reproductive lives. The proportion of individuals with one or more partners did not differ between males and females. Nine females had only one partner during their tenure, and 12 females had more than one. Ten males had only one partner, whereas 15 males had more than one (G-test, G_corr_ = 0.04, df = 1, p = 0.85). Males and females with more than one partner had on average 2.2 partners. Since these estimates were obtained using censored tenure length, the actual proportion of individuals that never had more than one partner during the entire life is probably even lower.

**Figure 1:**
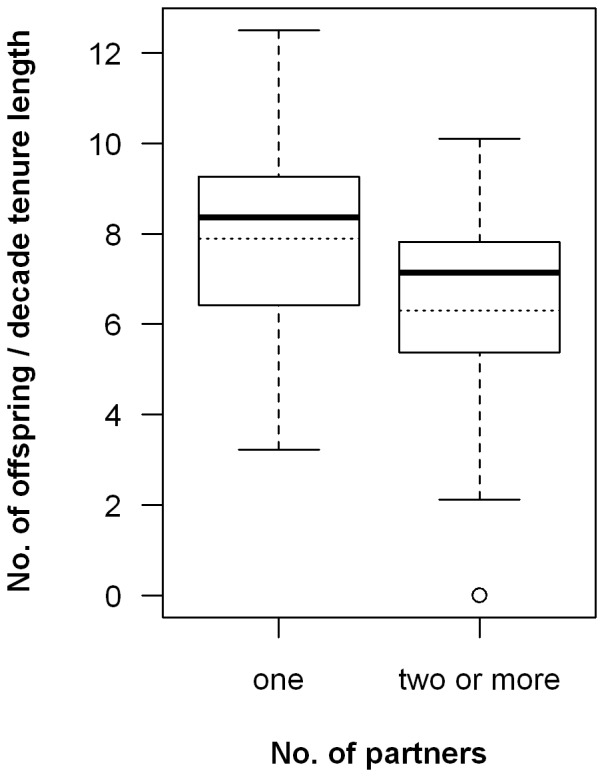
Number of infants per decade of tenure length (infants/10ytl). The boxplot show medians (solid line), means (dotted line) and interquartile ranges for individuals with one or with two or more partners during their tenure. The whiskers give the range except for “outliers” that are more than ±1.5 times the inter-quartile range larger or smaller than the median.

The change of partner reduced the reproductive success of the remaining individual. On average, an owl monkey who only had one partner produced 25% offspring more per decade of tenure than an individual with two or more partners (7.9 vs 6.3, glm, N_1_ = 19, N_≥2_ = 27, t = −2.1, p = 0.038; Fig. 1). This result was not significantly different for the sexes (glm, t = 0.42, p = 0.67). The difference in reproductive success was the consequence of a delay in reproduction following the formation of a new pair. The median inter-birth interval (IBI) of established pairs was 13.1 months (N = 22 pairs, 59 IBIs), whereas the mean delay between pair formation and first birth was 15.5 months (median: 14.4, N = 15 pairs). Since births are seasonal [16], when a pair dissolved, the remaining partner skipped a year of breeding, while stable pairs normally reproduced once a year. Only four of 22 pairs produced an offspring within the first year of pair-formation.

**Figure 2:**
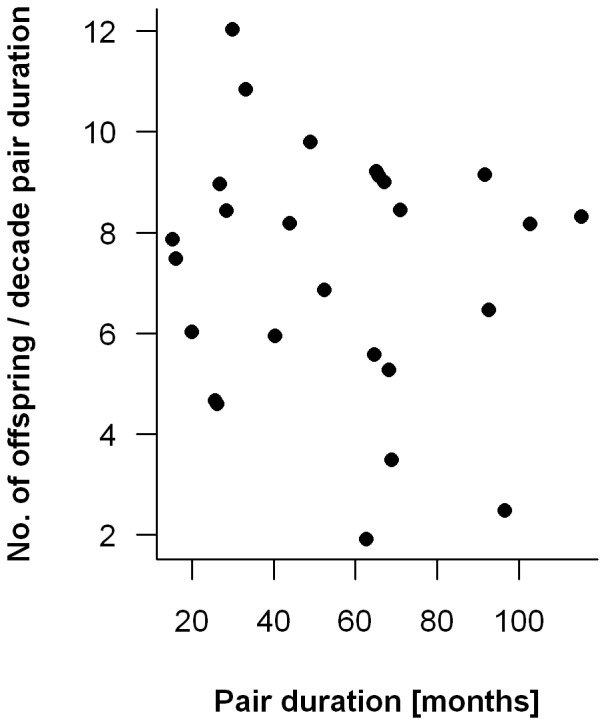
Correlation between the number of offspring per decade of pair duration and the total length a pair lasted.

There were no intrinsic factors clearly associated with pair-bond termination. Shorter pair durations were not associated with pairs that had fewer offspring per year (linear mixed model, t = −0.25, p = 0.81; Fig. 2), nor were break-ups more common after particularly long inter-birth intervals. Indeed, the median time between the birth of the last infant and break-up was 10.1 months, shorter than the average inter-birth interval reported above, not longer as would be expected if break-ups were triggered by failures to reproduce.

**Figure 3:**
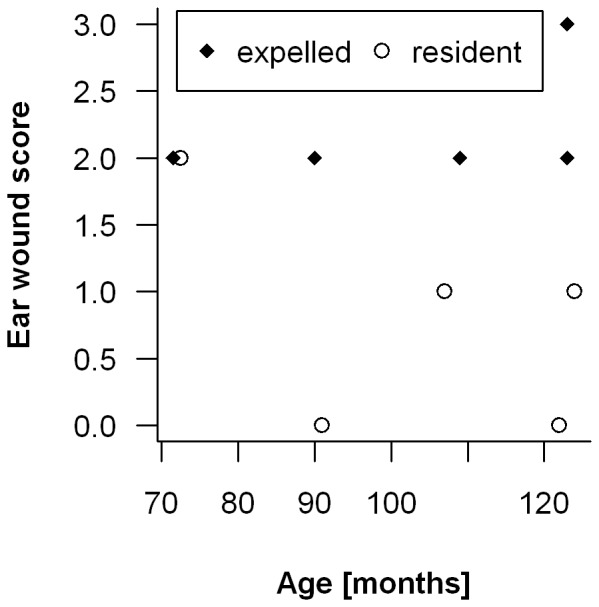
Ear wound score for adult owl monkeys. Ages for individuals that were still resident (♦) in a breeding group or recently expelled (○) are matched.

On the other hand, break-ups were strongly associated with factors extrinsic to the pair. In a majority of cases with detailed known circumstances (63%, 12/19 cases), one of the former residents was clearly expelled by the intruding individual. Among these cases there were five (four females, one male) when we either witnessed a fight resulting in wounds and limping, or we found the expelled individuals limping or with serious bite wounds shortly after the replacement; in one case this resulted in the death of the animal one day later. In about a third of the cases (37%, 7/19), the death of a former resident preceded the intrusion of a new individual, and never did a male or a female leave their partner (i.e., “divorced”). Secondary dispersal is infrequent; only two males of 50 expelled individuals successfully immigrated for a second time into a new group. Non-widowed owl monkey pairs clearly terminated their bond following the aggressive intrusion of a new individual. Ear wounds were more frequent among expelled individuals than among resident ones; all five expelled individuals showed ear wounds whereas only sixty-eight percent of 74 adults still resident as breeders had damaged ears. Furthermore, the degree to which ears were wounded differed markedly between residents and expelled individuals (average scores of 2.2 and 1.3 respectively; Mann-Whitney U-test: W = 279, N_1_ = 5, N_2_ = 72, p = 0.04), a difference that persisted when comparing residents and expelled individuals of matched age (Wilcoxon matched-pair test: W = 23, N = 5, p = 0.025; Fig. 3). There were no pronounced differences between the sexes in the average ear-wound scores (median_female_ = 2.0, median_male_ = 2.0; Mann-Whitney U-test: W = 669, N_female_ = 41, N_male_ = 36, p = 0.45).

## Discussion

Life-time reproductive success is highest for owl monkeys that do not change partners during their lifetime. A 25% increase during the life-time reproductive period should be a strong selective force. There could be other possible costs, potentially associated with the replacement of one of the parents, like reduced infant survivorship or early dispersal of juveniles and subadults. However, in our population, infant survival and age of dispersal did not change following replacements [28]. Our findings differ from those reported for some monogamous birds, where remaining life-time reproductive success (i.e., the expected future gains) of the individual that initiates or tolerates a ‘divorce’ was higher than if it remained with its initial partner. For example, in kittiwakes (*Rissa tridactyla*) and many other pair-living birds, but also in some human societies, it is sometimes advantageous to ‘divorce’, if partners prove incompatible [25], [27], [35]. In contrast, our data strongly indicate that break-ups were associated with factors extrinsic to the pair, and that partners did not voluntarily leave or “divorce” as it has been reported for birds, gibbons, and (in at least one case) brown titi monkeys (*Callicebus brunneus*) [25]–[27], [36], [37]. On the other hand, in some species (oystercatchers, *Haematopus ostralegus*), the reproductive success of stable pairs is not only higher, but there are also accrued benefits with increased duration of the pair-bond, independent of effects of age or experience [38]. This was not the case for owl monkeys, since the number of offspring produced did not change with increased duration of the pair-bond (Fig. 2).

Our results show that the owl monkey social system can be described as long-term serial monogamy. What remains to be carefully examined is the extent to which partners who have an interest in long-term stable bonds help his or her mate to repel intruders. We have shown that competition for breeding positions can be surprisingly fierce, sometimes leading to the death of one of the contestants. Thus, it is possible that the loss of fitness due to one missed breeding season is still lower than the potential costs of severe injuries during fights.

Similar findings of replacements through intense intra-sexual aggression have also been anecdotally reported for titi monkeys, another primate whose social organization is most similar to owl monkeys [37], [39]. These data also support earlier findings that, despite the relatively smaller canines of *Aotus* compared to most other primate species with high levels of intra-sexual aggression, the canines are substantially larger than those of closely related species with low intensity levels of aggression [7]. Monomorphism in this socially and genetically monogamous, bi-parental-care species might therefore be due to intra-sexual competition in both males and females, rather than to a lack of competition [7]. High potential levels of aggression among females, as well as among males are also found in gibbons (*Hylobates muelleri*) [15]. While it has been suggested that aggression among female birds might be over resources rather than mates [40], the replacements in owl monkeys seem to indicate that they are due to competition, for mates and a territory, at similar rates in females and males. Aggression has been shown to be beneficial in the context of female-female competition in other taxa as well, such as tree swallows (*Tachycineta bicolor*), European starlings (*Sturnus vulgaris*), and chimpanzees (*Pan troglodytes*) [41]–[43].

In populations where defensible territories are stable over some extended time, individuals are expected to fight fiercely over them since access to territories will highly affect their reproductive success [44]. The pressure to hold a territory should be particularly high if the survival of floaters is strongly reduced compared to group-living territory holders. As we have shown, all these conditions hold in our owl monkey population. Thus, we argue that our findings raise the question of whether the operational sex ratio, both theoretically and empirically linked to the degree of intra-sexual competition [1], [2], [6], can be assumed to be 1∶1 in pair-living species, even if males and females have similar potential reproductive rates. Rather, our data show that at each replacement event, several floaters of the same sex that roam solitarily, and without fixed home-ranges among the territories of established pairs, will compete for reproductive positions. In our population, both females and males can be affected by this process; but not necessarily at the same time or location (i.e., group). Thus, depending on whether we estimate the OSR for the whole population, only for established pairs, or for one particular group, we may conclude that the OSR is balanced, male-biased or female-biased.

In many species, it is very difficult to assess the floater population, or how many same-sexed individuals without established territories might at a given time compete with each other, since floaters tend to behave less conspicuously than established groups (pers. obs.). The potential presence of floaters of both sexes, and the subsequent context-specific OSRs that will result from their presence are usually neglected in sexual selection models, even when the influence of density-effects on the evolution of aggressiveness are well known [4]. In conclusion, given that it has long been recognized that OSRs can be time and place specific [6], [45], [46], the incorporation into sexual selection models of flexible, context-specific OSRs may help explain why many monogamous species are monomorphic, but, at least occasionally, also ferociously aggressive competitors.
